# Effects of Oral, Vaginal, and Transdermal Hormonal Contraception on Serum Levels of Coenzyme Q_10_, Vitamin E, and Total Antioxidant Activity

**DOI:** 10.1155/2010/925635

**Published:** 2010-08-09

**Authors:** Prabhudas R. Palan, Felix Strube, Juraj Letko, Azra Sadikovic, Magdy S. Mikhail

**Affiliations:** Department of Obstetrics and Gynecology, Bronx-Lebanon Hospital Center, Albert Einstein College of Medicine, 1650 Grand Concourse, Bronx, NY 10457, USA

## Abstract

The use of the transdermal contraceptive patch is associated with greater bioavailability of ethinyl estradiol (EE) compared with contraceptive vaginal ring or oral contraceptives (OC). We compared the influences of three contraceptive methods (OC, vaginal ring, and transdermal patch) on serum levels of coenzyme Q_10_, *α*-tocopherol, *γ*-tocopherol and total antioxidant capacity in premenopausal women. Blood samples from 30 premenopausal women who used hormonal contraception for at least 4 months were collected. Forty subjects who did not use any contraception were studied as control. Serum levels of coenzyme Q_10_, *α*-tocopherol and *γ*-tocopherol were measured by high-pressure liquid chromatography. Serum samples were also assayed for total antioxidant capacity (TAOC). Serum levels of coenzyme Q_10_ and *α*-tocopherol were found to be significantly lower (*P* < .05) in all three contraceptive users compared with controls. Contraceptive patch users had the lowest levels of coenzyme Q_10_ levels compared with normal subjects. Serum TAOC levels were significantly lower (*P* < .05) among the contraceptive user groups. Alterations in coenzyme Q_10_ and *α*-tocopherol induced by hormonal contraception and the potential effect(s) of exogenous ovarian hormones should be taken into consideration in future antioxidant research.

## 1. Introduction

Free radicals and related species have attracted a great deal of attention in scientific research. It has been reported that an imbalance between the production of oxygen free radicals and serum levels of antioxidants can lead to cell oxidative stress and damage and consequent apoptosis as a result of the excessive oxidation of lipids, nucleic acids, and/or proteins [1, 2]. Oxidative stress has been suggested to be involved in the etiology of many chronic disease processes including cardiovascular disease, cancer, cataract, and aging [1, 3, 4].

Ovarian hormones, primarily estrogens, possess antioxidant properties and have been postulated to protect against cardiovascular disease (CVD) [5, 6]. The lipid-soluble antioxidant, coenzyme Q_10_ (CoQ_10_), and *α*-tocopherol act as free radical scavengers and may decrease the risk of CVD caused by oxidative stress [4, 5]. Relatively little research has been focused on the effect of hormonal contraceptives on lipid-soluble antioxidants. Moreover, differences in contraceptive content and delivery methods of ethinyl estradiol (EE) may affect women differently. The use of the transdermal contraceptive patch is associated with greater bioavailability of estrogen than the use of the contraceptive vaginal ring or the oral contraceptive pill [6, 7]. We compared the influences of three different hormonal contraceptive methods (OC, the vaginal ring, and the transdermal patch) on serum levels of CoQ_10_, *α*-tocopherol, *γ*-tocopherol, and TAOC in premenopausal women. Such a three-way comparison of hormonal contraception could provide an insight into the effect of different contraceptive formulations on lipid-soluble antioxidant levels.

## 2. Material and Methods

### 2.1. Subject Population

The study protocol was approved by the Institutional Review Board. All subjects came from the same catchment area (Bronx, Borough of New York City), had similar inner-city socioeconomic background, and were healthy and not taking any antioxidant supplements. Women consuming CoQ_10_ or vitamin E supplementation were excluded from the study.

 In this cross-section study, 70 nonsmokers, healthy premenopausal women were recruited who attended GYN clinics at the Bronx-Lebanon Hospital Center, Bronx, New York. Of these 70 subjects, forty (*n* = 40) women who did not use hormonal contraceptive constituted the control group. Among the 30 contraceptive users: 15 took the oral pill (*Triphasic OC)* for a minimum period of 6 months; 5 inserted the vaginal ring *(NuvaRing)* from day 1–21 for a total period of 6 cycles; and 10 used the transdermal patch (*Ortho Evra)* for 3 consecutive weeks each month for a minimum of 6 cycles.

### 2.2. Patient Disposition

We studied three contraceptive delivery systems: OC, vaginal ring, and transdermal patch. The oral preparation studied was *Triphasic OC* containing 0.05 mg levonorgestrel + 0.03 mg EE for the first 6 days, 0.075 mg levonorgestrel + 0.40 mg EE for the following 5 days, and 0.125 mg levonorgestrel + 0.030 mg EE for the remaining 10 days. The vaginal delivery system *NuvaRing* releases 0.120 mg of etonogestrel and delivers 15 mcg of EE/day. The transdermal preparation *Ortho Evra* contains norelgestromin 6.0 mg and 0.75 mg EE, delivering 20 mcg of EE and 150 mcg of norelgestromin per day [6, 7].

### 2.3. HPLC Analysis of Serum Antioxidants

A peripheral venous blood sample (10 ml) was collected from each study subject between days 17 and 20 of the use of pill, patch, or ring during the sixth cycle. No dietary restrictions were imposed upon any of the subjects and patients were excluded if they were using any vitamin E or CoQ10 supplementation. A brief dietary questionnaire was completed by each participant. Serum was separated by centrifugation within 1-2 h after being drawn and stored at −80°C prior to analysis for no more than 7 days. Serum levels of CoQ_10_, *α*-tocopherol, and *γ*-tocopherol were measured by HPLC methods, as described previously [8, 9]. The coefficients of variation were <8% for all nutrients. Serum total cholesterol level was measured by the RIA method in the clinical laboratory at our institution.

### 2.4. Total Antioxidant Capacity Assay (TAOC)

TAOC was measured colorometrically with a commercially available kit (Northwest, Vancouver, WA) on a Luminometer [10]. The assay measures the ability of total antioxidant status of the serum to prevent the formation of peroxyl free radicals generated by thermal decomposition of 2,2′-azobis (2-amidinopropane) (ABAP). These peroxyl radicals react with luminol to generate a luminol radical (LH*) that results in emission of blue light centered at ≈425 nm. When antioxidants are present, luminescence is inhibited until the antioxidants are exhausted with the degree of suppression of color production being proportional to the antioxidant concentration of the added sample. Trolox, a water-soluble vitamin E analogue, was used as the standard, and the results were calculated in mM Trolox equivalents (mM Trolox equivalents/L). The analytical sensitivity of this method has been found to be 0.04 mM Trolox equivalents (*n* = 5) for serum. The intra-assay coefficient of variation was <2%.

### 2.5. Data Analysis

The data were expressed as means ± SD. Statistical analyses were performed using the Student *t* *-*test and ANOVA between (a) the contraceptive nonusers and users, and (b) the TAOC different between the four groups. Differences were considered statistically significant at *P* < .05.

## 3. Results

In this preliminary study, a total of 70 non-smoker women (median age: 33 y; range: 28–44 y) were enrolled with informed consent. The majority of study subjects were Hispanics (59%) and African-Americans (38%) and most of them were representatives of an inner-city underserved population.

Significantly decreased serum levels of CoQ_10_ and *α*-tocopherol (*P* < .001 by the Student's *t* *-*test) were detected in hormonal contraception users compared with nonusers (Table 1). The contraceptive patch users had the lowest serum levels of CoQ_10_. Serum levels of *γ*-tocopherol were comparable between OC users and controls. The CoQ_10_/cholesterol indexes were significantly lower in the subjects who used hormonal contraception compared to nonusers, demonstrating the lowest in the patch users.

The mean TAOC concentration in serum was 1.3 ± 1.0 mmol Trox equiv/l in the control group and the mean values of TAOC in the contraceptive pill, ring, and patch users were 1.00 ± 0.41, 0.85 ± 0.05, and 0.65 ± 0.04 mmol Trox equiv/l, respectively (Table 1). The lowest TAOC level was observed in the transdermal patch group and was found to be statistically significant compared with pill users (*P* < .05).

## 4. Discussion

This is the first report examining the effects of exogenous ovarian hormones on serum levels of CoQ_10_ and vitamin E in healthy premenopausal women. Results demonstrate significantly lower serum levels of CoQ_10_, *α*-tocopherol, and TAOC in hormonal contraception users compared to nonusers. Moreover, contraceptive patch users had the lowest levels of CoQ_10_ and TAOC. Data suggest that alterations in CoQ_10_ and *α*-tocopherol by hormonal contraception and the potential effect(s) of exogenous ovarian hormones on oxidative stress should be taken into consideration in future antioxidant research.

Of the three contraceptive formulations studied, the transdermal patch had the strongest lowering effect on CoQ_10_ levels and TAOC. The transdermal patch has been shown to produce a steady-state of EE concentrations of 58–71 pg/ml and maximum serum concentrations of 74–96 pg/ml [6, 7, 11]. The pharmacokinetics of the transdermal patch suggests that serum EE concentrations are higher than might be expected based solely on the amount of EE delivered [7]. Exposure to EE was previously reported to be highest for patients using the transdermal patch. The mean area-under-the curve was 3.4 times higher in the patch group than in the NuvaRing and 1.6 times higher than in the OC group [7]. It may be suggested that the greater bioavailability of EE reported with the transdermal patch may be responsible for the greatest lowering effect on serum CoQ_10_ levels and TAOC observed in our study.

Vaginal administration of contraceptive hormones allows low, steady, and continuous dosing and results in stable serum EE levels. The benefits of this low, precise dosing include lower systemic exposure to EE and a low incidence of estrogen-related side effects. Minimizing exposure to EE is desirable as it reduces estrogen-related side effects. NuvaRing is a monthly contraceptive vaginal ring. It works by releasing a continuous low dose of estrogen and progestin, on average 0.120 mg of etonogestrel and 0.015 mg of ethinyl estradiol per day over the period of use. NuvaRing has been shown to produce a mean serum EE concentrations of 19 pg/ml and maximum serum concentrations of 35 pg/ml [6, 7, 11]. Estrogen levels from the contraceptive vaginal ring (NuvaRing) are the lowest in any combined hormonal contraception currently available, and peak levels of ethinyl E2 are significantly lower in NuvaRing users than transdermal patch or oral contraceptive users [7, 11, 12]. Lastly, OC users have the greatest degree of variation in ethinyl E2 serum concentrations [7].

The present three-way comparison in this study of different hormonal contraceptives with different routes of administration and antioxidant status has not been previously reported [6, 7]. We previously described the effects of menopause and hormonal replacement therapy on decreased serum CoQ_10_ and *α*-tocopherol levels [9]. In this study, we examined the influence of different hormonal methods on lipid-soluble antioxidants in hormonal users.

 CoQ_10_ is regarded as one of the most important antioxidants. CoQ_10_ can be obtained from dietary sources of meat, fish, vegetables, and fruits. A typical Western diet provides 3 to 5 mg of CoQ_10_ daily, of which approximately two-thirds are derived from meat and poultry [13]. Tissues with high energy requirements, such as the heart, kidney, liver, and skeletal muscle contain high amounts of CoQ_10_ [14]. *α*-Tocopherol is generally considered the most potent antioxidant in the active tocopherols [15]. Vitamin E can be obtained from dietary sources of almonds, egg yolks, leafy vegetables, sunflower seeds, vegetable oils, and wheat germ. CoQ_10_ and *α*-tocopherol are lipid-soluble free radical scavengers located in cell membranes capable of neutralizing oxygen free radicals.

Oxidative stress is implicated in the pathogenesis of many diseases [2, 3, 16]. The reduction-oxidation (redox) state constitutes a potential mechanism for the regulation of many metabolic processes through modulation of signaling pathways. Oxidative reactions are an essential part of several biological systems and can have toxic effects depending on a critical balance between the oxidative stimulus and the antioxidant defense mechanisms available [1]. The imbalance between excessive redox generation and decreased antioxidant capacity leads to oxidative stress [16, 17]. It has been reported that an imbalance in the production of oxygen free radicals and the natural protective antioxidants can lead to oxidative stress-induced cell damage as a result of the excessive oxidation of lipids, nucleic acids, and proteins, and consequent apoptosis and cell loss [2, 3]. Antioxidants work cooperatively in biological systems and it is important to be able to correlate antioxidant measurements with antioxidant defenses and disease prevention. It is therefore recommended to study “total antioxidant capacity”, rather than monitoring individual antioxidant levels, which may be less affected by dietary habits; [18, 19] hence the measurement of TAOC in our study. There has been increasing interest in measuring the protective antioxidant activity of a variety of biological fluids and tissues. Most assays of TAOC utilize *in vitro* models of oxidative stress to which biological samples are exposed and the time to onset or the extent of oxidation is used to indicate total antioxidant capacity. TAOC appears to represent a mixed antioxidant response, rather than response to a single antioxidant [18–20]. While being affected by oxidative stress, the mechanism of the response may differ between clinical situations, such that the clinical significance of changes in serum TAOC remains to be defined.

While estrogen is known to possess antioxidant properties [21, 22], its use has been reported to be associated with decreased levels of some lipid-soluble antioxidants [22, 23]. We previously reported that oral contraceptive users had significantly lower plasma concentrations of B-carotene compared with intrauterine contraceptive device or barrier method users [23]. B-carotene is another potent lipid-soluble antioxidant. Our present study validates the potential reducing effect of hormonal contraceptives on lipid-soluble antioxidants. Since hormonal contraception is used by millions of women worldwide and decreased antioxidant levels have been associated with various chronic diseases, the potential reducing effect(s) of hormonal contraception on lipid-soluble antioxidants warrant further investigation.

The clinical relevance of differences in antioxidant profile and responses between transdermal, vaginal, and oral delivery is not known. Our findings demonstrate varying effects of exogenous ovarian hormones and their method of delivery on lipid-soluble antioxidant levels. All three methods of delivery (oral, vaginal, and transdermal) were associated with decreased CoQ_10_ and *α*-tocopherol levels and TOAC. The transdermal patch was associated with the lowest levels of CoQ_10_ and the maximum reduction in TAOC. The greater bioavailability of EE with the patch may contribute to the present observation. If our findings are confirmed by larger studies, women using contraceptives may be considered for CoQ_10_ and/or *α*-tocopherol supplementation. Further research is needed to investigate the potential value, if any, for CoQ_10_ and *α*-tocopherol supplementation in hormonal contraceptive users and the effect of the menstrual cycle phase on oxidative stress and antioxidant levels.

## Figures and Tables

**Table 1:** Age, weight, and serum levels of total cholesterol, coenzyme Q_10_, *α*-tocopherol, and *γ*-tocopherol, the ratios of coenzyme Q_10_/cholesterol and *α*-tocopherol/cholesterol in women who used the contraceptive pill, vaginal ring, or transdermal patch and control subjects.

	Contraceptive	Contraceptive users		
	Nonusers	Pill	Ring	Patch
Variables	Group 1 (*n* = 40)	Group 2 (*n* = 15)	Group 3 (*n* = 5)	Group 4 (*n* = 10)

Age (y)	35.8 ± 7.1	31.9 ± 5.6	33.5 ± 3.5	30.1 ± 7.6
Weight (lb)	146 ± 15	138 ± 13	124 ± 10	138 ± 19
Total Cholesterol (mmol/L)	4.86 ± 1.5	4.30 ± 0.8	4.54 ± 09	4.54 ± 0.9
TAOC (*n* = 5) (mmol Trolox equi./L)	1.33 ± 1.2	1.00 ± 0.41^b^	0.85 ± 0.05	0.65 ± 0.04
Coenzyme Q_10_ (*μ*mol/L)	0.69 ± 0.2^a, b^	0.44 ± 0.1^b^	0.39 ± 0.1	0.30 ± 0.1
*α*-Tocopherol (*μ*mol/L)	14.2 ± 3.0^a, b^	10.9 ± 2.1	10.9 ± 2.6	10.2 ± 1.6
*γ*-Tocopherol (*μ*mol/L)	2.66 ± 1.2	2.92 ± 0.72	3.28 ± 1.9	3.01 ± 0.7
CoQ_10_/Cholesterol (pmol/*μ*mol)	143.0 ± 47^c^	102.3 ± 26	85.9 ± 16	66.0 ± 20
*α*-Toco/Cholesterol (nmol/*μ*mol)	2.9 ± 0.6	2.5 ± 0.5	2.4 ± 0.6	2.6 ± 0.4

Values are mean ± SD.

^a^ *P* < .001, group 1 versus 2, 4; and ^b^ *P* < .05, group 1 versus 3; group 2 versus 4; and ^c^ *P* < .05, group 1 versus 2, 3, 4, by Student's *t*-test.
